# Roles of Specialized Pro-Resolving Lipid Mediators in Autophagy and Inflammation

**DOI:** 10.3390/ijms21186637

**Published:** 2020-09-10

**Authors:** Antonio Recchiuti, Elisa Isopi, Mario Romano, Domenico Mattoscio

**Affiliations:** Center for Advanced Studies and Technology, Department of Medical, Oral and Biotechnology Sciences, University of Chieti—Pescara, 66100 Chieti, Italy; a.recchiuti@unich.it (A.R.); e.isopi@unich.it (E.I.); mromano@unich.it (M.R.)

**Keywords:** autophagy, inflammation, chronic inflammation, resolution, specialized pro-resolving lipid mediators, resolvins, lipoxins, protectins, maresins, cystic fibrosis

## Abstract

Autophagy is a catabolic pathway that accounts for degradation and recycling of cellular components to extend cell survival under stress conditions. In addition to this prominent role, recent evidence indicates that autophagy is crucially involved in the regulation of the inflammatory response, a tightly controlled process aimed at clearing the inflammatory stimulus and restoring tissue homeostasis. To be efficient and beneficial to the host, inflammation should be controlled by a resolution program, since uncontrolled inflammation is the underlying cause of many pathologies. Resolution of inflammation is an active process mediated by a variety of mediators, including the so-called specialized pro-resolving lipid mediators (SPMs), a family of endogenous lipid autacoids known to regulate leukocyte infiltration and activities, and counterbalance cytokine production. Recently, regulation of autophagic mechanisms by these mediators has emerged, uncovering unappreciated connections between inflammation resolution and autophagy. Here, we summarize mechanisms of autophagy and resolution, focusing on the contribution of autophagy in sustaining paradigmatic examples of chronic inflammatory disorders. Then, we discuss the evidence that SPMs can restore dysregulated autophagy, hypothesizing that resolution of inflammation could represent an innovative approach to modulate autophagy and its impact on the inflammatory response.

## 1. Introduction

Autophagy is a physiological process that regulates the degradation and recycling of waste materials and damaged intracellular components through lysosomal activities. Initially described as a catabolic pathway activated under stress conditions and aimed to extend cell survival [1], a large body of evidence indicates now that autophagy is crucial for a variety of biological processes ranging from cell metabolism, organ development, differentiation, host defense, immunity, and inflammation. As such, alteration of the autophagic pathway has been correlated with several human pathologies like viral infections, neurological disorders, senescence, cancer, and inflammatory conditions [2]. In particular, the role of autophagy as modulator of inflammation, and its involvement in chronic inflammatory diseases is now emerging [3,4,5,6]. Indeed, autophagy may act as protective mechanism in the removal of viruses and bacteria, and a failure in the clearance of pathogens sustains the persistence of inflammation. In addition, a defective autophagic pathway fails to remove unnecessary cellular components, resulting in cell dysfunction and death that act as an inflammatory trigger. Importantly, autophagy regulates the production of inflammatory mediators, thus acting as an anti-inflammatory pathway in certain conditions (reviewed in [7]), and affects the activity of key immune cells such as polymorphonuclear leukocytes (PMNs) and macrophages (MΦs).

Acute inflammation is a protective response of vascularized tissues to injuries, traumas, or infections, which involves a tightly regulated and temporally restricted interaction and activation of endothelial and blood cells, as well as the production of specific chemical signals like cytokines and chemokines [8]. The physiological outcome of the inflammatory response is the restoration of tissue homeostasis and functionality, culminating in tissue repair. On the contrary, an uncontrolled inflammatory reaction can be detrimental and is indeed a driving pathogenetic mechanism for a wide range of human diseases [9]. To keep inflammation within physiologic boundaries, a proper resolution program must occur [10]. Resolution of inflammation is indeed an active process modulated by a variety of mediators, among which the so-called specialized pro-resolving lipid mediators (SPMs) play a preeminent role. These are endogenously-produced molecules that modulate leukocyte infiltration and activities, and cytokine release to terminate inflammation [11]. In addition, accumulating evidence indicates that modulation of autophagy is a relevant mechanism by which SPMs terminate the inflammatory response and restore tissue homeostasis. Failure in resolution has been associated with several diseases [12], suggesting that therapeutic induction of resolution may be proposed as an alternative approach to conventional anti-inflammatory pharmacology. Along these lines, SPMs proved beneficial in a several experimental diseases (reviewed in [13,14]) including periodontal and neurological diseases [15,16], arthritis [17], sickle cell disease [18], lung injury [19,20,21], and atherosclerosis [22]. Moreover, clinical studies with human volunteers confirmed that SPMs represent safe and effective therapeutics in asthma, skin infections, and eczema [23,24,25].

Here, we initially describe key mechanisms of autophagy and resolution of inflammation, focusing on the role of autophagy in chronic inflammatory disorders. Then, we discuss the emerging functions of SPMs as modulators of the autophagic pathway, and we outline our perspectives on the interplay between pro-resolving and autophagic pathways, proposing its modulation as innovative therapeutic strategy to combat human disease.

## 2. The Autophagic Pathway

The term autophagy (from Greek ‘to eat one’s self’) indicates several processes whose central function is to sequester cellular materials inside double-membraned organelles and to deliver autophagic cargoes to lysosomes for degradation.

In microautophagy, the digested materials are carried to lysosomes by direct protrusion or invagination of the lysosomal membrane to directly enwrap and degrade cytosolic components [26].

In chaperone-mediated autophagy, unfolded proteins that contain a specific pentapeptide motif are translocated by the heat shock cognate (hsc) protein 70 to the lysosome for degradation [27].

**Figure 1:**
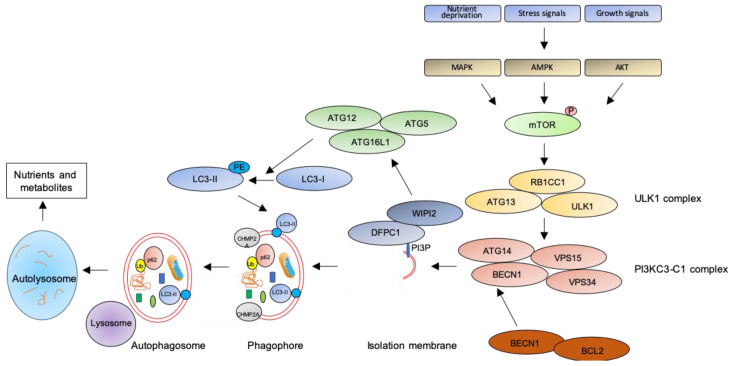
The autophagic pathway. See text for details.

In macroautophagy (simply referred as autophagy hereafter), the best studied autophagic route, a double-layered membrane named phagophore engulfs and encloses bulk cytoplasm or specific targets inside the autophagosome (Figure 1). The autophagosome then fuses with the lysosomal membrane where hydrolytic enzymes digest autophagosome cargo, and breakdown products are recycled back to cytosol for reuse [28]. Despite being initially considered as a non-selective pathway, induced as a survival mechanism in response to cellular stresses, it is now clear that autophagy is also a highly selective process involved in the clearance of protein aggregates (aggrephagy), intracellular pathogens (xenophagy), damaged or redundant organelles (such as pexophagy for peroxisome degradation, mitophagy for mithocondria, reticulophagy for endoplasmic reticulum, nucleophagy for nucleus, lysophagy for lysosomes), and macromolecular complexes (lipophagy for lipid droplets, ferritinophagy for ferritin, glycophagy for glycogen) (see [29] for a recent review on mechanisms of selective autophagy).

The key step of the autophagic pathway is the formation of the autophagosome, an endomembranous organelle de novo produced in stress conditions (readers are referred to [30] for a detailed review of the molecular mechanisms of autophagosome formation). The best characterized pathway of autophagy activation is regulated by the mechanistic target of rapamycin (mTOR) [31] that senses nutrient availability. Nutrients deprivation or other environmental factors, such as oxidative stress, inhibit mTOR through AMP-activated protein kinase (AMPK), protein kinase B (AKT), or mitogen-activated protein kinase (MAPK) activities, which phosphorylate mTOR and inactivate the mTOR1 complex (mTORC1). Inactive mTOR dissociates and activates the unc-51-like kinase 1 (ULK1) complex that, in turn, binds and activates the class III phosphatidylinositol 3–kinase complex I (PI3KC3-C1), which is recruited on the phagophore assembly site (initiation stage, see [32] for a comprehensive review of autophagy initiation). Here, the engaged complexes orchestrate the fusion of plasma membranes derived from endoplasmic reticulum, Golgi apparatus, or endosomes to finally generate the phagophore. In particular, the PI3KC3-C1 complex, that includes Beclin-1 (BECN1), autophagy-related 14 (ATG14), vacuolar protein sorting 15 (VPS15), and VPS34, locally produces phosphatidylinositol 3-phosphate (PI3P) (nucleation stage) [33] where lipid-binding proteins double FYVE domain-containing protein 1 (DFCP1) and WD repeat domain phosphoinositide-interacting protein 2 (WIPI2) are recruited and create a platform for assembly and activation of several autophagy-related proteins such as ATG12, ATG7, ATG10, ATG5, and ATG16L1, with an ubiquitin-like activity [34,35]. This sequential recruitment of autophagy factors on lipid domains initially produces the omegasome, membrane platforms connected to the endoplasmic reticulum [34], and culminates with the conjugation of another ubiquitin-like protein, microtubule-associated protein 1A/1B-light chain 3 (LC3-I), with the lipid phosphoatidylethanolamine (PE). Lipidated LC3 (LC3-II) is then anchored on the nascent phagophore where it mediates elongation of the phagophore membrane [36] (elongation stage). LC3-II is widely recognized as a key marker of autophagosome formation and autophagic activity, since lipidated LC3 targets autophagosome membranes and is then degraded after fusion with lysosome [37]. Finally, autophagosome closure is mediated by a component of the endosomal sorting complexes required for transport (ESCRT), CHMP2A (charged multivesicular body protein 2A), which regulates the formation of the double-membrane autophagosome through AAA-ATPase VPS4 activity [38]. The formed autophagosome sequesters cytosolic components and fuses with lysosomes (instigating the autolysosome) where autophagosome cargoes are exposed to the hydrolytic actions of lysosomal hydrolases (see [39] for a detailed review of autophagosome–lysosome fusion) (Figure 1). In selective autophagy, several adaptor proteins link payloads to the autophagosome, including the autophagic receptor p62 (or sequestosome 1, SQSTM1,) that binds cargoes (mostly ubiquitinated proteins) to LC3 in elongating phagophore [40]. Since p62 is itself degraded by autophagy, its expression levels are another canonical marker to monitor the autophagic flux [37]. For an efficient fusion, the autophagosome migrates to lysosome locations, predominantly found in perinuclear region, aided by the molecular adaptor Ras-related protein Rab-7a (Rab7a) that links the autophagosome to a microtubule motor [41]. The minus-end-directed dynein−dynactin motor complex transports the autophagosome close to a lysosome [42] where Rab proteins and tethering complexes drive cognate vesicle-soluble NSF attachment protein receptors (SNAREs) in autophagosome and lysosome to mediate the fusion of the two cellular compartments [43].

## 3. The Ideal Outcome of Inflammation: Resolution

### 3.1. Key Aspects of Acute Inflammation and Resolution

Acute inflammation is a tightly regulated and self-limited protective reaction against dangerous materials, expected to eliminate the insulting stimulus and to restore tissue function [44]. After injuries on vascularized tissues, the increase in endothelial permeability results in plasma leakage (edema) and a rapid PMN recruitment to remove the inflammatory stimulus by phagocytosis, degranulation, or formation of neutrophil extracellular traps (NETs) [45]. PMNs then undergo apoptosis and are removed through efferocytosis by MΦs differentiated from blood monocytes [46]. In addition to efferocytosis, MΦs are also able to remove the inflammatory trigger by non-phlogistic phagocytosis [47]. Successful efferocytosis then stimulates MΦ skewing from pro-inflammatory M1 to pro-resolving M2 phenotypes that dampen inflammation while stimulating tissue repair [48]. Once tissue homeostasis is restored, MΦs exit the inflamed tissue by the lymphatic system.

**Figure 2:**
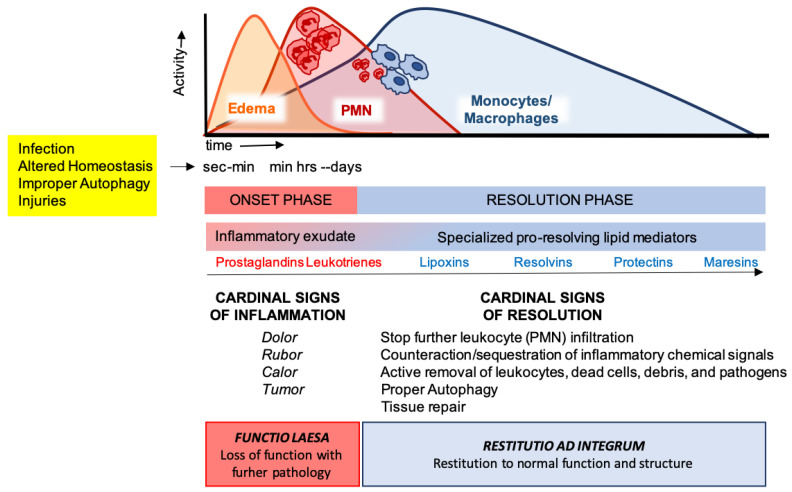
The acute inflammatory response and its ideal outcome: essential steps, mechanisms, and definitions. Injury, infections, or dysregulated homeostasis ignite the acute inflammatory response that is normally a host protective mechanism. The first event in acute inflammation is edema formation, followed by infiltration of polymorphonuclear leukocytes (PMNs), and then monocyte and macrophages that clear PMNs leading to resolution, which is essential for ensuring host protection and sparing from tissue damage.

These cellular events are timely and spatially regulated by the concerted production of proteins and lipids released by effector and resident cells [49]. Early in inflammation, pro-inflammatory eicosanoids (mainly prostaglandins-PGs and leukotrienes-LTs) derived from arachidonic acid (AA) metabolism), inflammatory cytokines and chemokines are produced by tissue-resident cells after sensing injuries like pathogen infection. These mediators in turn regulate blood flow, induce vasodilatation, edema formation and PMN infiltration, to mount the inflammatory reaction. Despite PMN recruitment is crucial to restrict the noxious stimulus, an excessive and uncontrolled PMN infiltration, activation and release of antimicrobial products may be harmful to the tissue as well as the prolonged secretion of pro-inflammatory mediators. Therefore, a resolution program should be started [50]. A crucial step in resolution is a lipid mediator class switch, from pro-inflammatory eicosanoids to SPMs that include lipoxin (LX), resolvins (Rv), maresins (Mar), and protectins (PD) (for a recent review on SPMs readers are referred to [51]). These are biosynthesized from essential polyunsaturated fatty acids (PUFAs) (see below) and act by limiting PMN infiltration, regulating the balance between pro-inflammatory and anti-inflammatory cytokines and chemokines, and by stimulating phagocytosis, efferocytosis, killing and wound healing by MΦs [11]. Notably, SPM biosynthesis can be modulated by pro-inflammatory mediators, for instance PGE_2_, generated during the onset of the inflammatory response [52], indicating that resolution is programmed from the beginning of the inflammatory reaction. Figure 2 illustrates an integrated view of inflammation and its resolution.

### 3.2. SPM Biosynthesis

Using a system approach of spontaneous and self-contained inflammation, pioneering studies from Dr Serhan’s laboratory lead to the identification of several previously undescribed lipid compounds in inflammatory exudates during the resolution phase [53]. These mediators act locally on different cell types by exploiting several receptors in a spatial- and temporal-dependent manner.

LXA_4_ and B_4_, the first SPMs identified [54], mainly derive from transcellular metabolism of AA involving PMN 5-lipoxygenase (LO) and platelet 12-LO [55,56]. In a second pathway of LX biosynthesis, AA is metabolized by the sequential activity of 15-LO in epithelial cells or MΦs and leukocyte 5-LO [57,58]. A third route of LX generation involves aspirin-acetylated cyclooxygenase (COX)-2 in vascular and epithelial cells and leukocyte 5-LO. LX produced by this metabolic pathway are isomers of native LX termed aspirin-triggered LX (ATL) A_4_ and B_4_ [59].

D-series Rv (RvD) encompass six metabolites (RvD1-6) of the omega-3 fatty acid docosahexaenoic acid (DHA) differing from number, position, and chirality of their hydroxyl residues, and position and isomerism of their double bonds [60]. The four members of E-series Rv (RvE1, RvE2, RvE3, and 18S-Rv1) are metabolites of the omega-3 fatty acid eicosapentaenoic acid (EPA) generated, similarly to ATL, through aspirin-acetylated endothelial COX-2 and PMN 5-LO transcellular activity [61]. More recently, the structure and biosynthesis of four members of the T-series Rv—which carry a 13-C position -OH group—have been elucidated. Specifically, similarly to RvE, RvT1-4 are produced from transcellular metabolism involving aspirin-acetylated endothelial COX-2 and PMN 5-LO but using n-3 docosapentaenoic acid (DPA) as precursor [62].

PDs are metabolites of DHA produced by PMNs, MΦs, and eosinophils, following enzymatic activity of 15-LO (PD1 or neuroprotectin when produced in neural system) or aspirin-acetylated COX-2 (AT-PD1) and subsequent enzymatic epoxidation and hydrolysis. PD1 isomerization leads to the production of a less-characterized PD member, PDX [63].

Mar1 and Mar2 are a third family of SPMs produced from DHA at sites of inflammation via a 12-LO enzymatic activity in MΦs [64], probably produced later during inflammation following the appearance of resolution phase MΦs.

In addition, recent work has uncovered additional SPMs showing potent tissue-protective activities in addition to their pro-resolution roles, and termed SPM conjugated in tissue regeneration (CTR). Characterization of their structural properties showed that CTRs originate from DHA and contain sulfido-conjugates derived from glutathione conjugation to DHA [65]. Maresins CTR (MCTR1-3) [66], resolvins CTR (RTCTR1-3) and protectins CTR (PCTR1-3) [65] are members of this class of SPMs. Finally, in addition to RvT, n-3 DPA is also the substrate of other SPMs with potent anti-inflammatory activities named PD1_n−3,_ RvD1_n−3_, RvD2_n−3_, RvD5_n−3_, MaR1_n−3_, MaR2_n−3_, and MaR3_n−3_ based on their structural analogies to PD, RvDs, and Mar, respectively [67].

### 3.3. SPM Receptors and Functions in Resolution of Inflammation

SPMs regulate inflammation and resolution via activation of different cell surface G-protein-coupled receptor (GPCRs) (reviewed in [68]), which rapidly transport signals and activate intracellular pathways to control a number of biological functions. Several SPM receptors have been identified to date mostly using library screening, labelled ligands for specific binding, GPCR–β-arrestin-coupled system and functional cellular responses involving gain and loss of function approaches [69,70,71].

Despite Formyl peptide receptor 2 (ALX/FPR2, also termed FPR2, FPRL1, or FPR2/ALX) being the first discovered and the most studied SPM receptor able to convey LXA_4_ bioactions in PMNs [72], it is now clear that this receptor is expressed in multiple cell types including myeloid cells and lymphocytes, resident endothelial, epithelial cells, fibroblasts [73], and stem cells [74], and is also activated by other SPMs (RvD1, AT-RvD1, RvD3, AT-RvD3, and AT-LXA_4_) [69,75,76,77,78]. In addition, in vitro studies showed that ALX/FPR2 is a promiscuous receptor that is also recognized and stimulated by a variety of natural and synthetic peptides to translate effects ranging from pro-inflammatory to anti-inflammatory and pro-resolution depending on the activating ligand (reviewed in [79]). However, the ALX/FPR2 transgenic and knock-out mouse models confirmed the crucial role of this receptor in inflammation resolution [80,81], and findings in human indicated that ALX/FPR2 levels dictate the amplitude of inflammation and its defective expression could be associated with inflammatory disorders [82,83].

In addition to ALX/FPR2, RvD1, AT-RvD1, RvD3, AT-RvD3, and AT-LXA_4_ also activate the human DRV1/GPR32 receptor [69,77]. Furthermore, RvD5 was also reported as agonist for DRV1/GPR32 [76].

RvD2 exerts its pro-resolving activities by binding and activation of the DRV2/GPR18 receptor in PMNs, monocytes and MΦs [84], thus stimulating bacterial clearance and organ protection in infectious inflammation [85].

ERV/ChemR23 is a GPCR very similar to ALX/FPR2 that binds and mediates RvE1 signals in monocytes, dendritic cells, and MΦs [70,71]. In addition, RvE1 also interacts with BLT1, the LTB_4_ receptor, as partial agonist, to dampen LTB_4_ pro-inflammatory signals while stimulating resolution through ERV/ChemR23 [86].

Similarly, Mar1 partially interacts with recombinant human BLT1 and acts as full agonist for the leucine-rich repeat containing G protein–coupled receptor 6 (LGR6) in MΦs to increase phagocytosis and efferocytosis of human and mouse phagocytes [87].

More recently, GPR37 was identified as potential receptor involved in PD1-mediated MΦs phagocytosis and resolution of inflammatory pain [88] and GPR101 proved to mediate pro-resolving actions of RvD5n-3 DPA [89].

On the other hand, if and how T-series Rv and the other 3-DPA metabolites and CTRs activate similar or different GPCRs is currently unknown.

At the molecular level, despite SPM/receptor interaction is cell- and organ-specific and influences a variety of pathways related to inflammation, some common features emerge. For example, most SPMs induce receptor dimerization, dampen nuclear factor kappa-light-chain-enhancer of activated B cells(NF-κB) [90] and Nrf-2 [18] activation, modulate the ERK, EGFR, and mTOR pathways [91], and regulate the expression of inflammatory microRNAs (miRNAs) [92]. At the cellular level, SPMs limit PMN recruitment and activity; enhance MΦ phagocytosis and efferocytosis; counter-regulate production of pro-inflammatory mediators, including PGs and LTs; chemokines and cytokines such as tumor necrosis factor α (TNFα), interleukin (IL)-6 and IL-8; growth factors (vascular endothelial growth factor, VEGF) and reactive oxygen species, whereas stimulating the production of nitric oxide (NO) and PGI_1_ by endothelial cells (recently reviewed in [93]). Since non-resolving inflammation underlies the pathogenesis of many human diseases and given their potent bioactions in cellular and preclinical models [12], SPMs are now being tested in selected human diseases with promising outcomes [23,24,25].

## 4. Autophagy in Chronic Inflammatory Diseases

### 4.1. Autophagy and Inflammation

Increasing evidence indicates that autophagy plays crucial roles in the regulation of the inflammatory response. Therefore, dysregulation of autophagy may contribute to trigger and amplify chronic inflammation and could be an underlying mechanism of human chronic inflammatory disorders.

As discussed above, the main function of autophagy is to maintain cellular functions under stress conditions, in order to prevent cell death. In addition to cell homeostasis, autophagy is centrally involved in host defense. Indeed, autophagy could be activated by invading microorganisms that trigger metabolic stress and inhibit mTOR, or by direct recognition of pathogens through innate immune receptors (toll like receptors—TLRs, NOD-like receptors—NLRs, and RIG-like receptors) or autophagic adaptors (sequestosome 1-like receptors—SLRs) [94]. Autophagy contributes to pathogens elimination by two mechanisms: sequestration and degradation of intracellular microorganisms by xenophagy [95]; decoration with LC3 molecules of pathogens captured by phagocytosis to promote phagosomal maturation and phagolysosomes formation (LC3-associated phagocytosis—LAP) [96].

Autophagy controls inflammation also by regulating the production of inflammatory mediators. The NLR family pyrin domain containing 3 (NLRP3) inflammasome is a multiprotein complex able to sense exogenous or endogenous damage and to produce the pro-inflammatory IL-1β and IL-18, thus inducing inflammation and pyroptosis [97]. Recent evidence shows that damaged mithocondria stimulate NRLP3 inflammasome activation [98] and that damaged mithocondia are removed by mithopagy [99]. Therefore, autophagy dysfunction impairs mithocondria removal, resulting in leakage of endogenous NLRP3 agonists, i.e., mithocondrial DNA and reactive oxygen species (ROS), and excessive production of IL-1β and IL-18 that sustain unrelenting inflammation [100]. Importantly, autophagy may down-regulate excessive inflammasome activity in the effort to dampen exaggerate inflammation [101], again suggesting that failure in autophagy may fail to counter-balance inflammation. In addition, autophagy negatively regulates type I interferon (IFN-I) production in response to DNA or RNA viruses (recently reviewed in [102]). For example, following detection of cytosolic viral DNA, BECN1, ATG9A, and ULK1 halt downstream signaling, leading to IFN-I production and anti-viral responses [103,104,105]. Similarly, viral RNA in cytosol stimulates RIG-I-dependent pathways that induce IFN-I production by mithocondria. This pathway is inhibited by the recruitment of ATG12-ATG5-ATG16L1 autophagic complex on mithocondrial membrane with suppression of ROS-mediated IFN-I release [106,107].

In addition to finely-tune the production of selected cytokines, autophagy may also regulate NF-κB activity, a key transcription factor of the inflammatory response [108]. Early work shows that p47 negatively regulates NF-κB activity by targeting NF-kappaB essential modulator (NEMO), a component of the IκB kinase (IKK) complex that stimulates NF-κB translocation to the nucleus [109], for lysosomal degradation [110]. Therefore, p47 emerges as an autophagic receptor to selectively promote NEMO degradation by autophagy. Furthermore, the F-box protein SKP2 acts as a bridge to promote the interaction between IKKb, a crucial kinase upstream NF-κB activation, and the autophagic receptor p62. This interaction finally leads to IKKb degradation via selective autophagy and reduction of NK-κB activity [111]. Similarly, the angiopoietin-like 8 (ANGPTL8) protein bridges IKKg with p62 and promotes IKKg degradation by selective autophagy, thereby negatively regulating NF-κB activation and inflammation [112]. Therefore, failure in selective autophagy may promote inflammation by circumventing regulatory loop that controls NF-κB activity.

Autophagy also crucially regulates important activities of PMNs and MΦs [113]. Indeed, autophagy is activated in PMNs and is necessary for PMN functions such as differentiation [114], bacterial clearance, degranulation and ROS production. Indeed, pharmacological and genetic approaches demonstrated that autophagy impairment reduces bacterial killing by human PMNs [115]. Along these lines, autophagy inhibition also reduces NETs formation [116], ROS production by inhibition of NADPH oxidase, and degranulation through altered fusion of PMN granules and phagosomes [117]. Collectively, these results suggest that autophagy inhibition may reduce the PMN-driven inflammatory reaction. Similarly, several monocytes/MΦs functions are crucially regulated by autophagy [118]. For instance, during monocyte-to-MΦ differentiation, ULK1 activation and BECN1 dissociation from inhibitory B-cell lymphoma 2 (BCL2) trigger autophagy and promote differentiation and generation of functional phagocytes [119,120]. In addition, autophagy regulates almost every aspect of MΦ biology, from pathogen recognition to cytokine release, inflammasome activation, and polarization. For further details please refer to a recent review on this area [121].

Moreover, autophagy plays critical functions in adaptive immunity. This topic has been discussed in some excellent reviews [7,122,123] and will not be further discussed here since it would be beyond the scope of this article.

Since autophagy is a key player of the inflammatory response, is not surprising that a number of chronic inflammatory human diseases are characterized by deregulated autophagy. Key aspects of some paradigmatic examples are listed below.

### 4.2. Chronic Inflammatory Diseases Characterized by Autophagy Dysfunction: Crohn’s Disease

Crohn’s disease (CD) was the first human pathology where a significant pathogenetic contribution of autophagy has been elucidated. CD is a chronic inflammatory bowel disease characterized by excessive inflammation and impaired bacterial clearance [124]. Genome-wide association studies revealed single nucleotide polymorphisms in autophagy genes, in particular ATG16L1, which is involved in autophagosome formation; immunity-related GTPase family M protein (IRGM) that regulates autophagy through mitochondria interaction; and the bacterial sensor, NOD2 [125,126]. Functional studies then revealed that defects in autophagy lead to impaired clearance of bacteria, altered production of pro-inflammatory cytokines [127], and defects in granule production by Paneth cells [128]. Importantly, Atg16l1 deficiency in mouse PMNs and MΦs increased ROS production but impaired microbial killing and altered IL-1β release of by MΦs [127,129], further documenting that defects in autophagy exacerbate bowel inflammation.

### 4.3. Chronic Inflammatory Diseases Characterized by Autophagy Dysfunction: Cystic Fibrosis

Autophagy dysfunction is critically involved in several pulmonary diseases characterized by non-resolving inflammation [130]. Cystic fibrosis (CF) is a genetic disorder caused by mutations (deletion of phenylalanine from the position-508—F508del is the most common) in the cystic fibrosis transmembrane conductance regulator (CFTR) gene [131], a membrane ion channel primarily regulating chloride efflux. Non-resolving lung inflammation in CF impairs host defense and is characterized by an uncontrolled recruitment of PMNs that, once infiltrated, release toxic products such as elastase, oxidant species, and NETs that cause tissue damage and mucus plugging. Evidence indicates that CFTR mutations stimulate a pro-inflammatory phenotype, even independently from infection [132]. Indeed, CF airways contain elevated concentrations of soluble mediators, including IL-8, -6, and LTB_4_ that contribute to the progression of lung disease [133]. Moreover, the reduced formation of SPMs may also contribute to CF relentless inflammation, suggesting that SPM administration could provide clinical benefits [19,20,21,134,135].

In addition to SPM defects, recent results point to autophagy as to a potential contributor to the pathogenesis of exuberant inflammation in CF. Indeed, human airway epithelial cells show accumulation of polyubiquinated proteins, impaired autophagy, and failure in the clearance of aggresome by aggrephagy. At the molecular level, defective CFTR causes the displacement of BECN1 from the endoplasmic reticulum and drives the sequestration of the PI3KC3 complex in aggresomes [120]. As a consequence, autophagy is disabled leading to defective autophagosome formation, accumulation of p62, and increased aggresome, which further sequester defective F508del CFTR [120]. Importantly, reconstitution of BECN1 restores CFTR trafficking to plasma membrane, reduces aggresome formation, and dampens production of pro-inflammatory mediators by CF cells, thus establishing a clear link between defective autophagy and sustained inflammation [136]. In addition, autophagic defects seem to be also related to the dysregulated NLRP3 inflammasome activity in CF. Indeed, administration of anakinra, an IL-1 receptor antagonist, to mouse and human CF models suppresses NLRP3-mediated inflammation and activates autophagy, suggesting that correction of autophagy could downregulate excessive inflammasome activity and thereby restore an appropriate inflammatory response [137]. Along these lines, treatment with the anti-inflammatory peptide IDR-1018 also attenuated hyperinflammatory cytokine secretion, restoring an appropriate autophagic flux in CF cells [138].

Importantly, rescuing autophagy also has beneficial effects against intestinal inflammation and dysfunction in patients with CF [139], thus extending the importance of autophagy as regulator of inflammation in multiple districts in CF.

In addition to epithelia, autophagy dysfunction also significantly contributes to defects of innate immunity in CF. Indeed, CF MΦs show weak autophagic activity, partially due to hypermetilation of the Atg12 promoter [140] and to elevated levels of the autophagy-regulating miRNAs miR-17 and miR-20a [141]. Stimulation of autophagy by the mTOR inhibitor rapamycin [142] or depletion of overexpressed p62 [143] reestablishes the ability of CF MΦs to clear *Burkholderia cenocepacia* infections and ameliorates lung inflammation. Similarly, autophagy inhibition improves the ability of mast cells to clear *Pseudomonas aeruginosa*, while pharmacologic induction of autophagy increases bacterial clearance in lungs of CF mice [144]. Along the same lines, treatment with the antibiotic azithromicin prevents lysosomal acidification and autophagosomal degradation, decreasing intracellular killing of multi-drug resistant *Mycobacteria abscessus* by MΦs, thus triggering chronic infection and inflammation [145]. Therefore, besides epithelia, autophagic deficiency also impairs the ability of immune cells to clear infections, thus sustaining chronic inflammation.

Collectively, these results indicate that CFTR deficiency causes an inhibition of autophagy in multiple cells and distinct organs in CF, suggesting that pharmacological induction of autophagy may provide benefits. Indeed, in vivo studies with CF mice, human CF primary cells, and patients with CF show that cysteamine plus epigallocatechin gallate (EGCG) restore BECN1-dependent autophagy, rescue functional F508del CFTR on the plasma membrane of nasal epithelial cells, and reduce pro-inflammatory cytokines in sputum of patients with CF [146,147]. These effects may be improved using the recently introduced combination cysteamine-amiodarone as autophagy inducers [148]. Other potential pharmacological treatments for CF based on autophagy correction were recently reviewed by Bodas et al. [149]. Although none of these treatments have yet reached the bedside, probably due to the high doses required and for the many off-target effects, approaches to restore autophagy and resolution of inflammation in CF can be potentially relevant.

### 4.4. Chronic Inflammatory Diseases Characterized by Autophagy Dysfunction: Cancer

Experimental and clinical evidence indicates that chronic, non-resolving inflammation contributes to the development and diffusion of neoplasms in humans [150,151]. Indeed, early events in tumor progression are characterized by altered production of inflammatory mediators and recruitment of inflammatory cells, i.e., PMNs and MΦ, to support tumorigenesis [151].

Likewise, altered autophagy is also a key driver of malignant transformation with a peculiar dual role depending on cancer stage. Indeed, in early steps of cancer development, autophagy works as a tumor suppressor pathway through removal of damaged organelles, proteins, ROS, and p62, which otherwise would sustain tumorigenesis and genomic instability. On the contrary, in established tumors, autophagy activation fuels the increased metabolic requirement of cancer cells by providing nutrients and energy that sustain tumor growth (recently reviewed in [152]). In addition, autophagy impacts on tumor development also by exploiting the inflammatory response to support the implant, growth, and pharmacological response of tumors.

For example, oncogenic viruses have developed clever mechanisms to revert control of cell proliferation and to trigger oncogenesis through manipulation of autophagy [153,154]. In fact, human papillomaviruses (HPV) down-regulate the autophagic response of infected keratinocytes in cervical and head and neck cancers to extend host’s cell lifespan [153]. In addition, recent work indicates that the HPV16 E7 oncoprotein manipulates autophagy to dampen the host immune reaction against infected cells. Indeed, E7 exploits autophagy to promote the degradation of STING, an immune signaling complex that stimulates anti-viral type I IFN production, thus impairing host immunity against HPV infections [155,156].

In addition, autophagic defects in mouse models of pancreatic cancer trigger the expression of pro-inflammatory cytokines such as Chemokine (C-C motif) ligand 5 (CCL5) and IL-6, and increase the infiltration of PMNs and T-cells, which fuel dysplastic transformation and tumor initiation [157]. Therefore, this observation indicates that autophagy restrains tumor induction by dampening inflammation. Along these lines, a proteomic investigation of Ras-driven cancer cells highlights that autophagy selectively targets specific inflammatory mediators such as IL-6, IFN-α, and IFN-β to suppress inflammation, and that autophagic defects activate innate immunity and the interferon response [158]. Consistent with this, autophagic-deficient tumor-bearing mice show increased expression of genes involved in the inflammatory response, leukocyte migration and immune cell trafficking as well as enhanced intertumoral MΦ infiltration, which sustain cancer progression and increase mortality [144,145]. Together, these results support the notion that defective autophagy in cancer promotes chronic inflammation that, in turn, sustains cancer development.

Importantly, autophagy and inflammation play a crucial role also during response to therapy. In fact, depletion of essential autophagic genes such as Atg5 or Atg7 in mouse allografts blunts the recruitment of immune cells, indicating that immunogenic response against cancer is crucially regulated by the autophagy-inflammation crosstalk [159,160]. Additionally, ATG7 siRNA in thyroid cancer cell lines sensitizes resistant cell lines to immune cell-mediated cytotoxicity [161]. Therefore, downregulation of autophagy during treatments may potentiate the immune reaction against residual tumor cells in selected cancers.

### 4.5. Chronic Inflammatory Diseases Characterized by Autophagy Dysfunction: Alzheimer’s Disease

Alzheimer’s Disease (AD) is one of the most common neurodegenerative disorder that is characterized by cognitive decline and the presence of amyloid β plaques and neurofibrillary tangles [162]. Over the last decade, a sustained immune response in the brain has emerged as a key pathogenetic feature in AD. Indeed, the chronic presence of highly insoluble deposits of amyloid β and tau and other inflammatory mediators may determine a functional impairment of microglia, the brain-resident MΦs-type cell, leading to chronic neuroinflammation and consequent neuronal degeneration and synaptic dysfunction [163]. Emerging evidence indicates that interplays between inflammation and autophagy may occur in the AD brain. Chronic inflammation determines intracellular ROS accumulation that, with aging, becomes progressively detrimental by triggering inflammasome formation [164], as well as apoptotic pathways and mitochondrial dysfunction [165]. These pathways induce autophagic mechanisms to suppress local damage [166]. More specifically, mitochondria impairment stimulates mitophagy that is impaired in the hippocampus of AD patients. Mitophagy stimulation reverses memory impairment through PINK-1 (PTEN-induced kinase-1), PDR-1 (Parkinson’s disease- related-1; parkin), or DCT-1 (DAF-16/FOXO-controlled germline-tumor affecting-1)-dependent pathways. Moreover, mitophagy diminishes insoluble Aβ1–42 and Aβ1–40 and prevents cognitive impairment in an APP/PS1 mouse model through microglial phagocytosis of extracellular Aβ plaques and suppression of neuroinflammation [167].

## 5. Stimulation of Resolution Restores Autophagy and Dampens Chronic Inflammation

It is now clear that the pro-resolving and the autophagic circuits can interact at several junctures, making the analysis of such interplay very appealing in the perspective of developing new and more potent anti-inflammatory pharmacology to combat a variety of chronic, life-threatening human diseases.

The first evidence that SPMs can activate autophagy comes from an elegant study showing autophagy induction by RvD1 and AT-LXA_4_ in human and murine monocytes and MΦs [168]. Treatment with nanomolar concentrations of these SPMs triggers LC3-I to LC3-II formation, increases autophagosome formation while decreasing p62 levels, suggestive of autophagy activation. Notably, RvD1 and AT-LXA_4_ activate autophagy through a MAPK1 pathway, independently from mTOR, by inducing the dissociation of BCL2 from BECN1, with subsequent BECN1 activation and autolysosome formation. Importantly, RvD1 and AT-LXA_4_ stimulate phagocytosis of zymosan particles by MΦs in an ATG5-dependent manner [168], suggesting that autophagy activation is crucial for the removal of inflammatory stimuli, to promote resolution and avoid chronic inflammation.

Along these lines, an agonist of the ALX/FPR2 receptor, BML-111, promotes LC3-II accumulation in alveolar MΦs primed with LPS to mimic acute lung injury, suggestive of increased autophagosome formation. In addition, reduction of p62 levels confirms that stimulation of ALX/FPR2 by BML-111 activates autophagic flux through a MAPK pathway independent from mTOR, suggesting late autophagic activation. Interestingly, BML-111 administration to rats with acute lung injury reduces the concentration of pro-inflammatory cytokines in lung lavage and activates autophagy in alveolar MΦs, supporting the hypothesis that enhanced autophagy dampens inflammation [169].

Another ALX/FPR2 agonist, RvD1, proved beneficial in restoring autophagic flux in a mouse model of cerulein-induced acute pancreatitis (AP), where autophagy plays a clear role in supporting inflammation [170]. Indeed, AP tissues are characterized by impaired autophagy with higher expression of BECN1, p62, LC3-II, and increased number of autophagic vacuoles. Treatment with RvD1 normalizes the autophagic flux in AP mice by reducing BECN1, p62, LC3-II levels, and the number of autophagic structures [171].

Similarly to RvD1, RvE1 has been recently reported as modulator of doxorubicin-induced autophagy in cardiac tissues. Indeed, administration of RvE1 to doxorubicin-treated mice restores normal levels of BECN1, p62, and LC3-II in heart by an AKT/mTOR-dependent signaling pathway, suggesting that RvE1 protects from cardiotoxicity by modulating autophagy [172].

Opposite to RvD1 and RvE1, LXA_4_ inhibits obesity-induced autophagy in mice fed with a high-fat diet [173]. Chronic obesity promotes excessive autophagy which, in turn, sustains cell death and adipose inflammation [174]. Treatment with LXA_4_ or its stable analog benzo-LXA_4_ increases p62 and reduces LC3-II expression in adipose tissue of high-fat diet animals as compared to standard-diet littermates, indicative of autophagic flux suppression. Importantly, this activation is independent from both mTOR and AMPK activity [173], suggesting that LXA_4_ may regulate a late stage of autophagosome formation, consistently with previous results [168]. Similarly to LXA_4_, Mar1 has been reported as an autophagy inhibitor in models of obesity. Indeed, treatment of 3T3-L1 adipocytes with a pro-inflammatory stimulus such as TNF-α reduces p62 and increases LC3-II levels, suggestive of autophagy activation, and these effects are reverted in a concentration-dependent manner by pre-treatment with Mar1 [175]. However, Mar1 effects on autophagy may vary with the biological setting. Indeed, in models of chronic periodontitis, Mar1 activates autophagy in human periodontal ligament cells (PDL) exposed to LPS, as suggested by the upregulation of LC3-II and BECN1, and the downregulation of p62 in Mar1-treated PDL [161]. Notably, the autophagic inhibitor 3-MA antagonizes the inhibitory actions of Mar1 on IL-6, IL-8, TNF-α, and IL-1β release by PDL exposed to LPS [176], suggesting that Mar1 limits inflammation by activating autophagy. Moreover, Mar1 has been reported to stimulate autophagy in a mouse model of AD, where in addition to improve cognitive decline and to balance the production of pro-inflammatory and anti-inflammatory mediators, it increases BECN1 and LC3-II levels and decreases p62 in the hippocampus, indicative of autophagy stimulation [177].

In addition to SPMs, some of their precursors proved effective in autophagy modulation. In particular, in hepatocytes primed with palmitate to mimic low grade chronic inflammation, lipotoxicity, and insulin resistance associated with metabolic disorders in obesity [178], the DHA-metabolite 17(S)-HDHA restores autophagy as demonstrated by LC3-II and p62 accumulation in cells with blocked autophagic flux [179].

**Figure 3:**
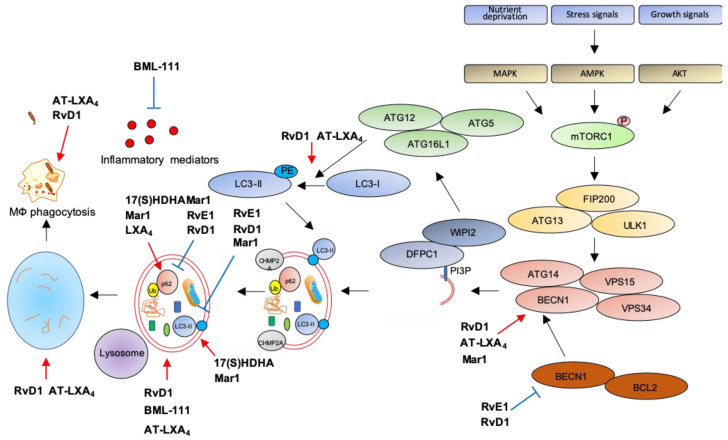
Modulation of autophagy by SPMs. Red arrows: increase; blue bar-headed arrows: decrease in indicated proteins or subcellular compartments.

The following Table 1 and Figure 3 summarize the role of SPMs as modulators of autophagy and inflammation in several models of human diseases.

## 6. Conclusions

The development of innovative pharmacology to target chronic inflammatory diseases is the object of intense research, because of the considerable impact that findings in this field can have on human health. The discovery of SPMs has opened intriguing avenues in the pharmacology of inflammation, providing new perspectives for simultaneously stopping excessive inflammation, promoting resolution and tissue repair by stimulating one’s body defense in a non-phlogistic manner. This is of utmost importance for the treatment of chronic inflammatory diseases, since traditional anti-inflammatory therapies deputed to only reduce the mounting of the inflammation response and also impair some relevant mechanisms that trigger the resolution phase. Therefore, despite their established value, currently anti-inflammatory drugs are associated with important side and immunosuppressive effects, which instead are not observed with SPMs [60]. In addition to their well-recognized role as modulators of inflammation and resolution, SPMs promote tissue remodeling, reduce pain, and carry anti-cancer properties by regulating several molecular and cellular pathways. Among these, autophagy is now emerging.

Autophagy is a cellular process initially considered to extend cell survival under stress conditions, now widely recognized as fundamental regulator of a variety of biological processes and is involved in a number of human pathologies characterized by unrelenting inflammation such as CD, CF, cancer, and AD. Therefore, modulation of autophagy may represent a promising strategy to combat a wide array of inflammatory disorders [4,5]. On the other hand, improper modulation of autophagy may be dangerous, underlying the need for well-targeted autophagy-directed therapies. In keeping with this, the emerging evidence of autophagy modulation by SPMs shows some promise.

**Figure 4:**
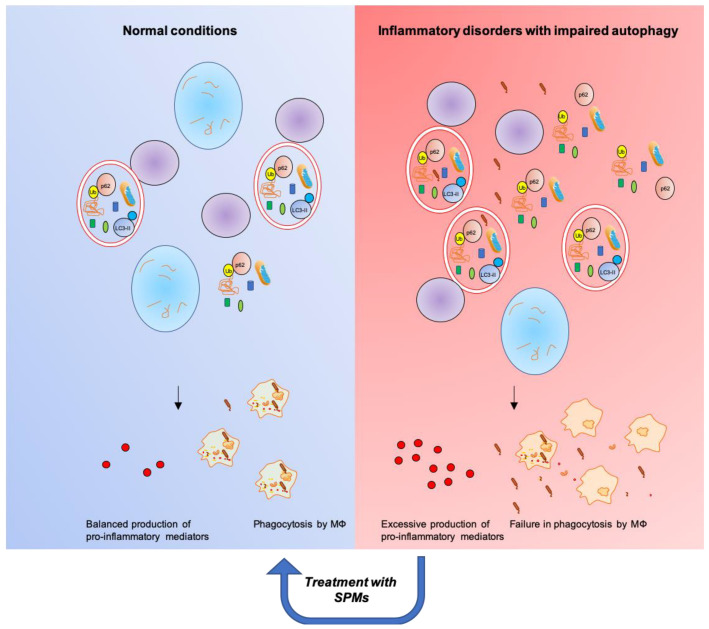
SPMs restore functional autophagic flux and resolution of inflammation in inflammatory disorders characterized by impaired autophagy.

Although different SPMs may have different bioactions on autophagic pathways, the emerging scenario is that SPMs predominantly activate autophagy in inflammatory conditions characterized by autophagy suppression (Table 1 and Figure 4). Under these circumstances, SPMs restore the autophagic flux by acting at multiple levels (Figure 3). In turn, autophagy stimulation by SPMs promotes MΦs phagocytosis and blunts the production of pro-inflammatory cytokines, both cardinal signs of resolution. On the contrary, in conditions characterized by a low-level of chronic inflammation [180] and sustained autophagy, such as obesity, SPMs appear to inhibit the autophagic response. Thus, SPMs known to promote inflammation resolution appear also to act as normalizers of the autophagic system, protecting the cell from excessively high or low activity of the system. These are relevant aspects within the field of inflammation and related disorders that deserve adequate investigation.

## Figures and Tables

**Table 1:** Role of SPMs as modulators of autophagy and inflammation in human diseases.

SPMs	Disease Model	Molecular Effect	Effect on Autophagy	Refs
RvD1 and AT-LXA_4_	MΦs inflammation	BECN1 dissociation and activation MAPK dependent, autolysosome formation	Activation	[168]
BML-111	Acute lung injury, impaired autophagy	LC3-II accumulation, p62 degradation MAPK dependent	Activation	[169]
RvD1	Acute pancreatitis, impaired autophagy	Reduction in BECN1, p62, LC3-II	Reactivation of impaired autophagic flux	[171]
RvE1	Cardiotoxicity, impaired autophagy	Reduction in BECN1, p62, LC3-II	Reactivation of impaired autophagic flux	[172]
LXA_4_	Obesity, increased autophagy	LC3-II decrease, p62 increase	Inhibition	[173]
Mar1	Obesity, increased autophagy	LC3-II decrease, p62 increase	Inhibition	[175]
17(S)-HDHA	Obesity, increased autophagy	LC3-II and p62 accumulation in autophagic flux experiments	Inhibition	[179]
Mar1	Periodontitis	Increase in LC3-II and BECN1, decrease in p62	Activation	[176]
Mar1	Alzheimer, impaired autophagy	Increase in LC3-II and BECN1, decrease in p62	Activation	[177]
